# Carditis Simulating Chorea

**Published:** 1829-10-01

**Authors:** 


### CARDITIS SIMULATING CHOREA.

The medical practitioner is not seldom puzzled by nervous affections taking on the characters of serious organic diseases:—he should be on his guard against the reverse—dangerous maladies concealed under the mask of nervous disorders. The following case is published in Hufeland's journal, by Dr. Roeser of Wurtemburg.

*Case.* A young girl, nine years of age, of delicate constitution, was seized with epistaxis, while at school, on the 25th November, 1827, and the blood continued to flow till syncope supervened. The hæmorrhage returned two days afterwards. She had never before experienced any illness. On the 1st December the mother applied to Dr. Roeser, on account of certain involuntary and convulsive movements which the child presented, especially about the head and in the limbs. These increased daily, and by the 7th of the same month, the girl could neither walk nor stand. The disease was now pronounced by Dr. R. to be chorea. The patient complained of pains in her head, in the region of the heart, and in her limbs. We need not detail the treatment. The poverty of the patient prevented much polypharmacy. We may remark, however, that, in addition to the usual choreal symptoms, there was complete insomnium—small and quick pulse—red sediment in the urine —yellow coat on the tongue. These adjuncts determined M. Roeser to adopt the purgative plan of Hamilton. It was, however, ineffectual, for in a very few days the patient died—not indeed before the doctor began to suspect that he had a disease to combat somewhat more formidable than St. Vitus's dance.

*Dissection.* There were marks of inflammation, and even considerable effusion in the head. But the chest presented the most remarkable lesions. The lungs were covered with gellatinous exudations, partly membraniform, though their substance was sound. The anterior mediastinum contained much of the same products of inflammation. The pericardium contained from four to five ounces of sero-sanguineous fluid—and it was connected by elongated bands of false membrane to the surface of the heart, which surface was completely coated with coagulable lymph, of several lines in thickness.

It need hardly be said that such a condition of the heart ought to have been recognized, to a greater or less extent, during life. But it seems that in Germany the value of auscultation is still less appreciated than in this country. Our Saxon brethren are too much taken up with the dreams and placid slumbers of animal magnetism, to be alive to the sober *revelations* of the senses, and the physical *signs* of the times. It may not be easy to imagine the kind of ecstasy experienced on awaking from such reveries to the sad realities presented by cases like the foregoing! We abhor that censorious spirit, now alas! too prevalent in the profession, which condemns, with uncharitable demeanour, every mistake of a neighbour; but we do think that it is incumbent on the medical practitioner to avail himself of every possible mean which may contribute to enlighten the dark and intricate paths he is obliged daily to tread.

